# Perceived community cervical cancer prevention support and cervical cancer screening utilization among women in Shenzhen, China: a cross-sectional study

**DOI:** 10.3389/fpubh.2026.1964812

**Published:** 2026-09-23

**Authors:** Meihua Wu, Lin Shen, Nan Jiang, Honggui Wang, Mei Wang, Yunyu Zhang, Qiuxiang Wu, Huijun Bai, Yan Ke, Wenming Cao

**Affiliations:** 1 Shenzhen Hospital of Integrated Traditional Chinese and Western Medicine, Shenzhen, China; 2 Pingshan District Central Hospital of Shenzhen, Shenzhen, Guangdong, China

**Keywords:** cervical cancer screening, community health services, cross-sectional study, health services research, primary health care, service utilization

## Abstract

**Purpose:**

Community health services have the capacity to inform eligible women about cervical cancer prevention, recommend screening, and assist with access to care. Whether favorable perceptions of community support are associated with completed screening in an urban Chinese setting is unclear.

**Methods:**

We conducted a cross-sectional electronic survey of women recruited through more than 20 community health service centers affiliated with two hospitals in Shenzhen and through gynecology outpatient clinics. The source dataset comprised 3,277 completed questionnaires: 2,142 (65.4%) recruited through community health service centers, 853 (26.0%) through gynecology outpatient clinics, and 282 (8.6%) through community outreach events. The analysis included 3,033 women aged 18–65 years. The outcome was self-reported cervical cancer screening within the preceding 3 years. A six-item perceived community cervical cancer prevention-support score was evaluated using Cronbach’s alpha and examined in multivariable logistic regression with sociodemographic and screening-related covariates.

**Results:**

Of 3,033 women, 1,314 (43.3%) reported screening within the preceding 3 years. The support scale had good internal consistency (Cronbach’s alpha = 0.918; mean 3.79, SD 0.83). In this cross-sectional sample, the overall support score was not independently associated with completed screening after adjustment (aOR = 0.935, 95% CI 0.856–1.021; *p* = 0.135). Knowledge of screening methods was associated with prior screening (aOR = 1.184 per one-point increase, 95% CI 1.102–1.271; *p* < 0.001). In exploratory item-level analysis, expected appointment support for vaccination or screening was inversely associated with prior screening (aOR = 0.881, 95% CI 0.790–0.983; *p* = 0.023). In exploratory item-level analysis with Benjamini-Hochberg false discovery rate correction for multiple testing, no individual community support item remained statistically significant.

**Conclusion:**

In this sample, favorable perceived community prevention support was not independently associated with completed cervical cancer screening. Future prospective studies that distinguish perceived support from documented service contacts, including invitations, bookings, attendance, result communication, and follow-up, are needed to evaluate whether active navigation pathways improve verified screening completion.

## 1 Introduction

Cervical cancer (CC) remains a preventable cause of illness and premature death, yet the preventive pathway fails when vaccination, screening, diagnosis, and treatment do not reach women in a timely sequence (1). In 2022, an estimated 660,000 new cases and 350,000 deaths occurred worldwide, with the greatest burden concentrated in settings where organized prevention and treatment pathways remain incomplete (2). The World Health Organization (WHO) has framed CC elimination around three linked targets: 90% human papillomavirus vaccination coverage among girls, 70% screening coverage among women by 35 and again by 45 years of age, and 90% access to treatment for women with cervical disease (3). Screening is therefore not an adjunct to prevention (4, 5). It is one of the operational conditions required for elimination.

China has expanded CC prevention through public screening programs, primary-care services, and HPV vaccination initiatives (6). In China, cervical cancer screening (CCS) has been delivered through a combination of organized public programs and opportunistic screening in clinical settings (7, 8). Opportunistic screening, where testing is offered to women presenting for other reasons, may reach women already engaged with health services but can leave behind those with limited access or lower health-seeking behavior (4, 8, 9). Urban community health services may contribute at several points in the screening pathway: they can identify eligible women, communicate where screening is available, provide clinician recommendation, assist with booking, and link women to follow-up services (10–12). The significance of these functions is predicated on the understanding that screening is not a discrete decision. It is imperative that women are able to recognize their eligibility for the relevant tests, and that they have a comprehensive understanding of the available options. Furthermore, they must be able to locate an appropriate service, arrange a visit, attend the examination, receive the result, and, when necessary, complete the referral process (2). WHO has also emphasized that screening programs require organized pathways for invitation, testing, communication of results, and treatment, rather than testing capacity alone (3, 13).

Research in the field of cervical cancer screening (CCS) has historically focused on a range of factors including knowledge, beliefs, practical constraints, clinician contact, and access to care (8). However, the exposure and outcome definitions employed across studies are not uniform (14). It is evident that a range of assessments are employed to measure awareness of prevention. For instance, some assessors utilize questionnaires to document general awareness, while others employ more rigorous methods by measuring stated intention. Additionally, some studies employ a combination of both methods, documenting both general awareness and stated intention. Finally, some studies also inquire about the completion of screening procedures, providing a more comprehensive approach to measuring awareness and prevention practices (15). These are related but not interchangeable stages of service use. WHO guidance places screening within an organized pathway that extends beyond information provision to invitation, testing, communication of results, and treatment (3).

An unresolved issue concerns the meaning of perceived community support in settings where health information and local services are already visible (16). Favorable ratings of community promotion, recommendation, or service accessibility may indicate a supportive environment (17). They may also coexist with an incomplete pathway from awareness to attendance (18). Moreover, women who have not been screened may report a stronger anticipated need for appointment help than women who have already completed screening (19). This possibility is especially relevant in cross-sectional surveys, in which perceived support and prior behavior are assessed at the same time (20). Evidence is therefore needed on whether perceived community screening support distinguishes women who have completed screening from those who have not, after accounting for sociodemographic characteristics and screening-related knowledge and beliefs (21).

Shenzhen provides a useful urban setting in which to examine this question. The city has a large and mobile population, extensive community health infrastructure, and substantial variation in educational, employment, and insurance circumstances within a shared metropolitan service environment (22). A focus on service utilization also separates the present study from work on HPV vaccination willingness or hesitancy (23). The relevant outcome here is completed CCS, not intention alone.

This study examined whether perceived community CC prevention support was associated with self-reported screening completion within the preceding 3 years. We distinguished perceived support from documented service delivery and treated the analysis as cross-sectional, without presuming a causal pathway from support to screening behavior. By distinguishing perceived support from completed service use, this study addresses a practical question for community prevention programs: whether favorable perceived conditions are associated with screening completion, or whether further attention should be directed toward measurable steps such as invitation, appointment scheduling, attendance, result communication, and follow-up.

## 2 Methods

### 2.1 Study design and setting

This was a cross-sectional analysis of an electronic community survey conducted in Shenzhen, China, between August 2023 and April 2026. The study examined self-reported CCS service utilization and perceived community support among women living in Shenzhen. The reporting of this observational study was guided by the Strengthening the Reporting of Observational Studies in Epidemiology (STROBE) statement (24).

### 2.2 Participants, recruitment, and data collection

Participants were recruited using a non-probability convenience sampling approach. A questionnaire was created on the *Wenjuanxing* online survey platform^1^, which generated a unique quick-response (QR) code and survey link. The QR code and link were distributed to women through more than 20 community health service centers affiliated with two hospitals in Shenzhen. Recruitment also occurred among women attending gynecology outpatient clinics for CCS at the participating hospitals. The source dataset comprised 3,277 completed questionnaires: 2,142 (65.4%) were recruited through community health service centers, 853 (26.0%) through gynecology outpatient clinics at participating hospitals, and 282 (8.6%) through community outreach events associated with these facilities. Recruitment source was included as a covariate in all adjusted models, and stratified analyses by recruitment setting were conducted as sensitivity analyses; the association between the community support score and screening completion was not statistically significant in any subgroup (all *p* > 0.10).

Potential participants accessed the questionnaire by scanning the QR code with a mobile telephone. The first page of the survey provided study information and an electronic informed-consent statement. Only respondents who consented to participate were permitted to proceed to the questionnaire. The survey collected a range of data including sociodemographic characteristics, knowledge of CC prevention, screening-related beliefs, perceived social and community support, screening behavior, screening intention, and information preferences. Participants completed the questionnaire independently on their mobile devices.

In China, CCS recommendations vary by region. The Chinese Preventive Medicine Association and the Chinese Society of Gynecologic Oncology recommend that sexually active women aged 25–64 years undergo routine screening (25). Given that our research question concerned utilization of adult CCS services among women targeted for prevention interventions, we included women aged 18–65 years (26). This broader range encompasses younger women (18–24 years) who may be candidates for HPV vaccination, and older women (65 + years) for whom prior screening history remains relevant to understanding preventive service engagement (27). All participants were recruited from community health service centers and gynecology outpatient clinics, settings where CCS is typically offered to sexually active women. A sensitivity analysis restricted to the guideline-recommended age range of 25–64 years yielded results consistent with the primary analysis (aOR = 0.932, 95% CI 0.851–1.021; *p* = 0.128).

The source dataset comprised 3,277 completed questionnaires. For the present analysis, women aged 18–65 years were eligible for inclusion in the study because the research question concerned the utilization of adult CCS services. Respondents under the age of 18 (*n* = 62) or over the age of 65 (*n* = 182) were excluded from the study. The final analytic sample comprised 3,033 women. In this age-eligible sample, all variables included in the primary regression model were complete; no additional observations were excluded on the basis of missing data (Figure 1).

**Figure 1:**
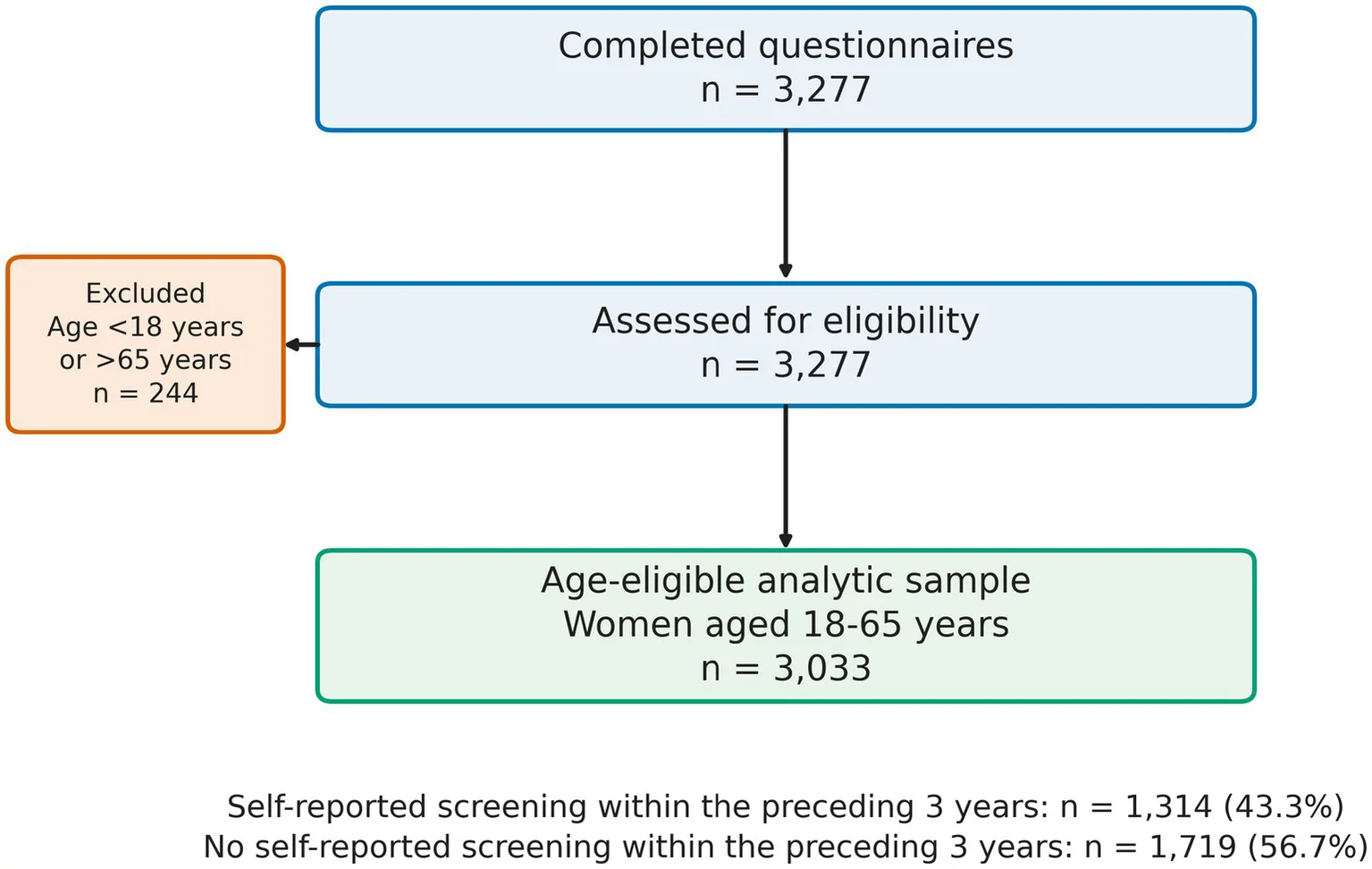
Participant flow diagram. The source dataset comprised 3,277 completed questionnaires. Respondents aged <18 years (*n* = 62) or >65 years (*n* = 182) were excluded from the analysis as the focus was on adult CCS service utilization, resulting in an analytic sample of 3,033 women. No observations were removed due to missing values in the primary model. Screening completion was defined from self-report of CCS during the preceding 3 years. The flow diagram is provided as an SVG vector file for publication-quality scaling.

Because recruitment was conducted through community health services and gynecology outpatient clinics, the sample should not be interpreted as a probability sample of all women in Shenzhen. The design may preferentially include women who had access to participating health facilities, were able to use a mobile telephone, or were already engaged with health services.

### 2.3 Outcome: CCS service utilization

The primary outcome was self-reported completion of CCS within the preceding 3 years. The original questionnaire classified respondents into three categories: screened within the preceding 3 years; not screened but planned screening during the current year; and not screened with no current-year plan. For binary logistic regression, the outcome was coded as 1 for women reporting screening during the preceding 3 years and 0 for the two non-screened categories combined. Women who were attending clinics for screening at the time of recruitment were classified according to their reported history of screening in the preceding 3 years; the screening visit at recruitment was not counted as prior screening because the questionnaire assessed history before the current visit.

The three-category screening-status variable was retained for descriptive and group-comparison analyses because it distinguishes completed service use from current intention and from no stated plan. Screening intention was additionally described among women who had not completed screening during the preceding 3 years using a five-point item ranging from very unwilling to very willing to complete screening during the current year. Neither the screening-status categories nor intention responses were treated as longitudinal transitions or as evidence of future screening completion.

### 2.4 Perceived community cervical cancer prevention support

The primary exposure was perceived community CC prevention support. Six questionnaire items assessed whether: (1) the community frequently promoted CC prevention knowledge; (2) a community doctor had recommended HPV vaccination or CCS; (3) the respondent knew that community CCS services were available; (4) health information provided by the community was trustworthy; (5) community staff would assist with appointment booking for vaccination or screening if needed; and (6) the scheduling of community vaccination services was reasonable. Each item used a five-point Likert response scale from 1 (strongly disagree) to 5 (strongly agree).

The questionnaire was developed based on a review of the literature on cervical cancer screening determinants and community health service utilization. Items were generated by the research team, which included epidemiologists, public health researchers, and clinicians with expertise in cervical cancer prevention and community health services (19, 28). A preliminary version was pilot-tested with 50 women attending community health centers to assess clarity, comprehensibility, and face validity. Based on pilot testing feedback, items were revised for clarity. The final questionnaire was reviewed by three experts in cervical cancer prevention and community health for content validity. Exploratory factor analysis supported a single-factor structure (eigenvalue = 4.39, 73.2% variance explained; factor loadings 0.71–0.89), indicating unidimensionality of the six items.

The overall score was calculated as the arithmetic mean of the six items, with higher values indicating greater perceived support. Internal consistency was evaluated using Cronbach’s alpha. The scale was designed to reflect the integrated nature of CC prevention services in Chinese community health settings, where HPV vaccination and CCS are often promoted jointly through health education campaigns and clinical recommendations (29, 30). The score contains items that refer jointly to vaccination and screening and one item that refers specifically to vaccination-service scheduling; it was therefore interpreted as perceived community CC prevention support, rather than as a direct measure of delivered screening services. The six components were entered into an exploratory item-level logistic regression model, with the data being entered simultaneously. The objective of this analysis was to ascertain whether any component exhibited an association with previous screening that was distinct from the composite score. The analysis was not designed to establish the effect of delivered community services.

### 2.5 Covariates

The sociodemographic covariates included in the study were age group (18–26, 27–37, 38–48, or 49–65 years; these categories were chosen to reflect meaningful life-stage transitions relevant to reproductive health and CC risk, to ensure adequate sample sizes within each group for stable estimation, and to align with conventions in previous Chinese CCS research), household registration status (Shenzhen or non-Shenzhen hukou), marital status (never married, married, or divorced/widowed), educational attainment, monthly income, health insurance type, and parity. The inclusion of household registration was driven by the potential influence of hukou status on access to local social and health services. Recruitment source (community health service center, gynecology outpatient clinic, or community outreach) was also included as a covariate in adjusted models.

The study incorporated five screening-related variables: knowledge of CCS methods; belief that regular screening enables early detection; embarrassment or discomfort related to screening; perceived lack of time for screening; and screening self-efficacy. Each participant was measured on a five-point response scale. Scores of higher magnitude are indicative of a greater endorsement of the named construct. These variables were selected because they represent knowledge, beliefs, and practical perceptions that may be associated with screening behavior and may also be related to perceived community support. They were treated as concurrent correlates rather than mediators, given the cross-sectional design.

### 2.6 Statistical analysis

Analyses were performed in the age-eligible analytic sample (n = 3,033). Categorical variables are reported as frequencies and column percentages. Continuous scale scores are summarized as means and standard deviations for descriptive presentation; screening-status comparisons of Likert-type variables are reported as medians with 25th and 75th percentiles.

Cronbach’s alpha was calculated to assess the internal consistency of the community CC prevention-support scale. Participant characteristics were compared across the three screening-status groups using Pearson chi-square tests. Cramer’s V was reported to describe the magnitude of categorical associations. No expected cell count was below 5. Kruskal-Wallis tests were used to compare ordinal Likert-type measures across the three independent screening-status groups. Effect size was calculated as eta-squared = H/(N-1), where H is the Kruskal-Wallis statistic and N is the analytic sample size. Values of 0.01, 0.06, and 0.14 were interpreted as small, medium, and large effects, respectively.

All analyses were performed using R version 4.3.2. The assumption of linearity in the logit for continuous predictors was assessed using Box-Tidwell tests and visual inspection of loess-smoothed plots; no evidence of nonlinearity was detected (all *p* > 0.05). Multicollinearity was assessed using variance inflation factors (VIFs); all VIFs were below 3, indicating no problematic collinearity.

The primary adjusted analysis used binary logistic regression, with self-reported screening within the preceding 3 years as the dependent variable. The overall community CC prevention-support score was entered as a continuous predictor. Sequential models were fitted: Model 1 (unadjusted), Model 2 (adjusted for sociodemographic characteristics only), and Model 3 (fully adjusted for sociodemographic and screening-related covariates). The model additionally included the five screening-related variables, all sociodemographic covariates, and recruitment source (community health service center, gynecology outpatient clinic, or community outreach). Categorical covariates were represented by dummy variables using the following reference categories: age 18–26 years, Shenzhen household registration, married, junior high school or below, monthly income <5,000 yuan, commercial health insurance, and nulliparity. Adjusted odds ratios (aORs), 95% confidence intervals (CIs), and two-sided *p* values are reported. Model fit was summarized using the likelihood-ratio chi-square test and Nagelkerke R-squared.

Because six support items were examined simultaneously in the item-level model, we applied the Benjamini-Hochberg false discovery rate (FDR) correction for multiple testing. Separate adjusted models for each item (excluding other support items) were also conducted as sensitivity analyses; no individual item was significantly associated with screening completion in these separate models.

To account for potential clustering at the facility level, we conducted multilevel logistic regression with random intercepts at the community health service center level; the intraclass correlation coefficient was 0.018, indicating minimal clustering, and the association remained non-significant (aOR = 0.931, 95% CI 0.849–1.021; *p* = 0.132; data not shown). Additionally, because the outcome prevalence was 43.3%, we performed robust Poisson regression to estimate adjusted prevalence ratios; results were consistent with the logistic regression findings (aPR = 0.981, 95% CI 0.951–1.012; *p* = 0.214).

A two-sided *p* value <0.05 was considered statistically significant. The analyses were complete-case analyses because no observations had missing data for variables included in the primary model.

All associations were interpreted as cross-sectional. In particular, perceived community support, anticipated appointment assistance, screening-related beliefs, and prior screening history were measured at the same survey time point. The analyses therefore cannot establish temporal sequence or causality.

### 2.7 Ethics statement

The study was approved by the Ethics Committee of Shenzhen Hospital of Integrated Traditional Chinese and Western Medicine (KY-2023-033-02) and the Ethics Committee of Pingshan District Central Hospital (PZXY-EC-AP-020). Electronic informed consent was obtained from participants before they accessed the questionnaire. The study was conducted in accordance with the ethical principles of the Declaration of Helsinki.

## 3 Results

### 3.1 Participant inclusion and analytic sample

The source dataset comprised 3,277 respondents. Following the exclusion of 244 respondents who were under the age of 18 or over the age of 65, the primary analysis was conducted on a total of 3,033 female participants (Figure 1). It was found that all variables necessary for the primary model were complete in the age-eligible sample. The Chinese source questionnaire and its English translation are provided in the Supplementary material S1.

### 3.2 CCS status and participant characteristics

Within the analytic sample, 1,314 women (43.3%) reported CCS during the preceding 3 years. Of the remaining women, 1,005 (33.1%) reported an intention to undergo screening within the current year and 714 (23.5%) reported neither prior screening nor a plan to screen. Thus, 56.7% of eligible respondents had not completed screening during the previous 3 years, despite 58.5% of this unscreened group reporting a current-year plan.

Marital status, educational attainment, monthly income, and insurance type differed across the three screening-status groups (Table 1). The corresponding Cramer’s V values ranged from 0.052 to 0.058, indicating small absolute differences. Age group, household registration status, and parity were not associated with screening status.

**Table 1:** Participant characteristics by CCS status (*n* = 3,033).

Variable	Category	No screening and no plan, *n* = 714	Not screened; plans screening this year, *n* = 1,005	Screened within previous 3 years, *n* = 1,314	Chi-square	*p-*value
Age group, (years)	18–26	189 (26.5)	284 (28.3)	391 (29.8)	4.886	0.558
	27–37	266 (37.3)	349 (34.7)	445 (33.9)		
	38–48	164 (23.0)	227 (22.6)	281 (21.4)		
	49–65	95 (13.3)	145 (14.4)	197 (15.0)		
Household registration	Non-Shenzhen hukou	349 (48.9)	467 (46.5)	664 (50.5)	3.769	0.152
	Shenzhen hukou	365 (51.1)	538 (53.5)	650 (49.5)		
Marital status	Never married	261 (36.6)	361 (35.9)	534 (40.6)	20.729	<0.001
	Married	405 (56.7)	563 (56.0)	641 (48.8)		
	Divorced/widowed	48 (6.7)	81 (8.1)	139 (10.6)		
Educational attainment	Junior high school or below	19 (2.7)	41 (4.1)	85 (6.5)	18.516	0.005
	High school/technical secondary school	116 (16.2)	180 (17.9)	209 (15.9)		
	College diploma	237 (33.2)	309 (30.7)	396 (30.1)		
	Bachelor’s degree or above	342 (47.9)	475 (47.3)	624 (47.5)		
Monthly income, (yuan)	<5,000	217 (30.4)	359 (35.7)	500 (38.1)	18.485	0.005
	5,000-9,999	237 (33.2)	322 (32.0)	443 (33.7)		
	10,000-19,999	184 (25.8)	227 (22.6)	266 (20.2)		
	> = 20,000	76 (10.6)	97 (9.7)	105 (8.0)		
Health insurance type	No medical insurance	54 (7.6)	81 (8.1)	81 (6.2)	16.237	0.013
	Resident medical insurance	245 (34.3)	362 (36.0)	409 (31.1)		
	Employee medical insurance	347 (48.6)	465 (46.3)	713 (54.3)		
	Commercial health insurance	68 (9.5)	97 (9.7)	111 (8.5)		
Parity	Nulliparous	302 (42.3)	420 (41.8)	574 (43.7)	0.905	0.636
	Parous	412 (57.7)	585 (58.2)	740 (56.3)		

Values are n (column %), with each percentage calculated using the screening-status group shown in the column header. Screening status was based on self-report: no screening and no current-year plan; no screening during the preceding 3 years but planned screening during the current year; and screening during the preceding 3 years. Household registration refers to Shenzhen hukou status. Pearson chi-square tests compare the three screening-status groups. Cramer’s V values were 0.028 for age, 0.035 for household registration, 0.058 for marital status, 0.055 for education, 0.055 for income, 0.052 for insurance, and 0.017 for parity; these indicate small absolute associations. No expected cell count was below 5.

### 3.3 Perceived community CC prevention support

The six-item community CC prevention-support scale had good internal consistency (Cronbach’s alpha = 0.918; Supplementary Table S1). The mean score was 3.79 (SD = 0.83) on the 1–5 response scale. Awareness of community screening services had the highest mean score (*M* = 3.90, SD = 0.94), whereas trust in community health information had the lowest (*M* = 3.57, SD = 0.99) (Figure 2).

**Figure 2:**
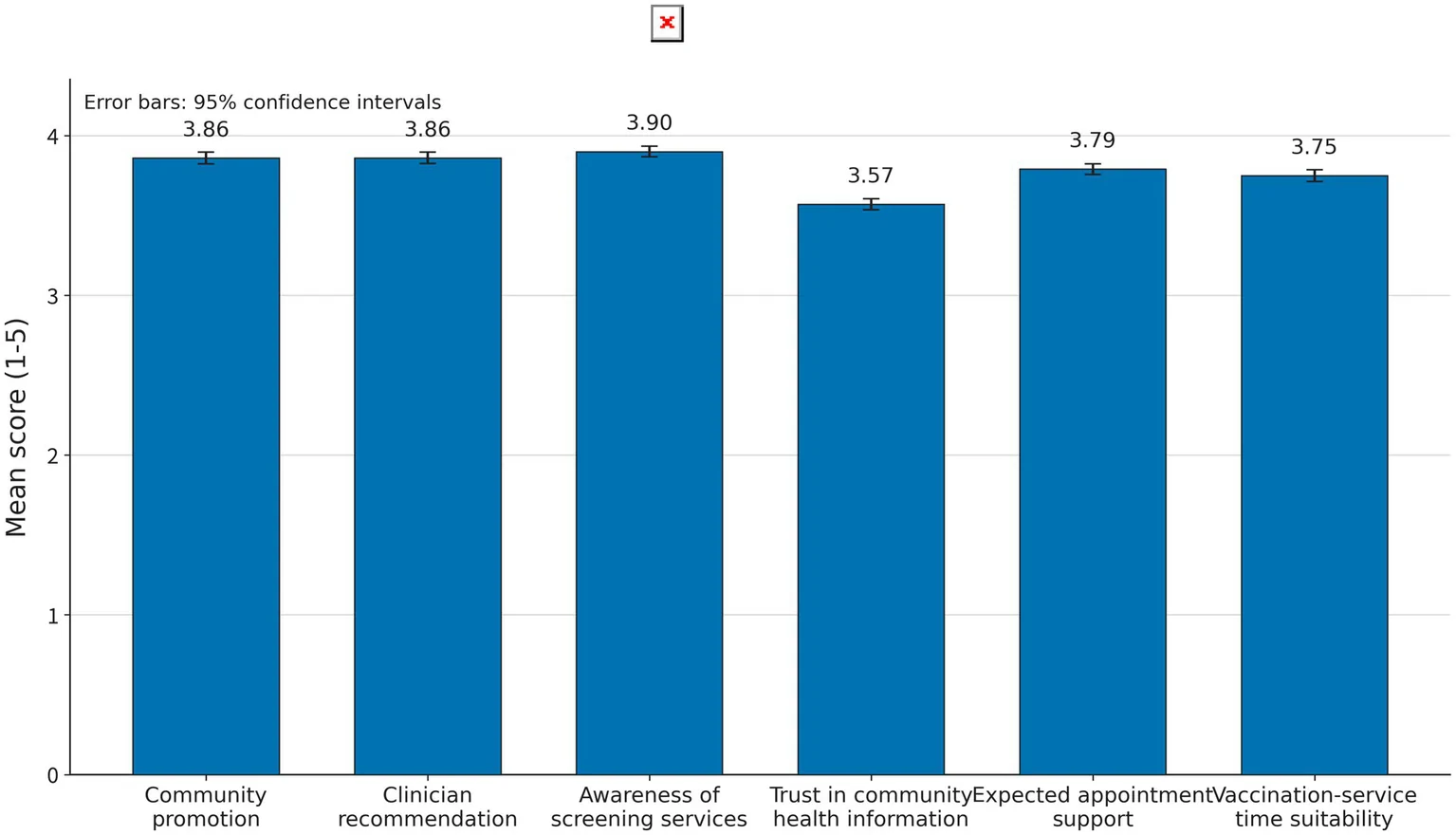
Mean scores for perceived community CC prevention-support domains. The six domains were rated from 1 (strongly disagree) to 5 (strongly agree); higher scores indicate greater perceived support. Bars show arithmetic means and error bars show 95% confidence intervals calculated from the age-eligible analytic sample (*n* = 3,033). Community health promotion, clinician recommendation, awareness of community screening services, trust in community health information, expected appointment support for vaccination or screening, and vaccination-service time suitability are presented in the order used in the questionnaire. A single blue hue, black text and axes, and a white background were used so that interpretation does not depend on red-green color contrast.

Community CC prevention support did not differ across the three screening-status groups (*H* = 2.883, *p* = 0.237). Scores for community promotion, clinician recommendation, service awareness, trust in community information, vaccination-service time suitability, and screening self-efficacy were likewise similar across groups. Appointment-support scores differed statistically (*H* = 13.022, *p* = 0.001), but the effect size was negligible (eta-squared = 0.004). Complete item-level group comparisons are provided in Supplementary Table S2.

### 3.4 Screening-related beliefs across the service-utilization continuum

Screening-method knowledge differed across groups (*H* = 59.795, *p* < 0.001), as did perceived screening benefit (*H* = 14.232, *p* < 0.001) and embarrassment or discomfort related to screening (*H* = 59.591, *p* < 0.001; Table 2). The associated effect sizes were small (eta-squared = 0.005–0.020). In contrast, perceived lack of time and screening self-efficacy did not differ across the service-utilization continuum.

**Table 2:** Composite community prevention support and screening-related factors by screening status (*n* = 3,033).

Domain	Measure	No screening/no plan	Not screened/plans this year	Screened within 3 years	*H*	*p-*value	Eta-squared
Community prevention support	Composite score	3.83 (3.33, 4.33)	3.83 (3.33, 4.50)	3.83 (3.33, 4.33)	2.883	0.237	0.001
Screening knowledge	Knowledge of screening methods	3.00 (2.00, 4.00)	3.00 (3.00, 4.00)	3.00 (3.00, 4.00)	59.795	<0.001	0.020
Screening belief	Screening enables early detection	4.00 (3.00, 5.00)	4.00 (3.00, 4.00)	4.00 (3.00, 4.00)	14.232	<0.001	0.005
Perceived barrier	Screening embarrassment/discomfort	2.00 (2.00, 3.00)	2.00 (2.00, 4.00)	3.00 (2.00, 4.00)	59.591	<0.001	0.020
Perceived barrier	Lack of time for screening	4.00 (3.00, 4.00)	4.00 (3.00, 4.00)	4.00 (3.00, 4.00)	0.592	0.744	<0.001
Self-efficacy	Ability to overcome barriers to screening	4.00 (4.00, 5.00)	4.00 (3.00, 5.00)	4.00 (3.00, 5.00)	5.270	0.072	0.002

All measures were rated from 1 to 5, with higher values indicating greater endorsement of the named construct; values are median (P25, P75). Kruskal-Wallis tests compared the three independent screening-status groups because the measures were ordinal Likert-type variables. Eta-squared was calculated as H/(N-1); values of 0.01, 0.06, and 0.14 are conventionally interpreted as small, medium, and large effects, respectively. The six individual community prevention-support items are reported in Supplementary Table S2. The data are cross-sectional, so observed group differences do not establish temporal ordering or causation.

Among women who had not completed screening during the previous 3 years, 966 of 1,719 (56.2%) reported being willing or very willing to complete screening during the current year (Figure 3 and Supplementary Table S3). A further 614 women (35.7%) reported a neutral intention, whereas 139 women (8.1%) reported unwillingness. These figures describe intention at the time of survey and do not represent future screening completion.

**Figure 3:**
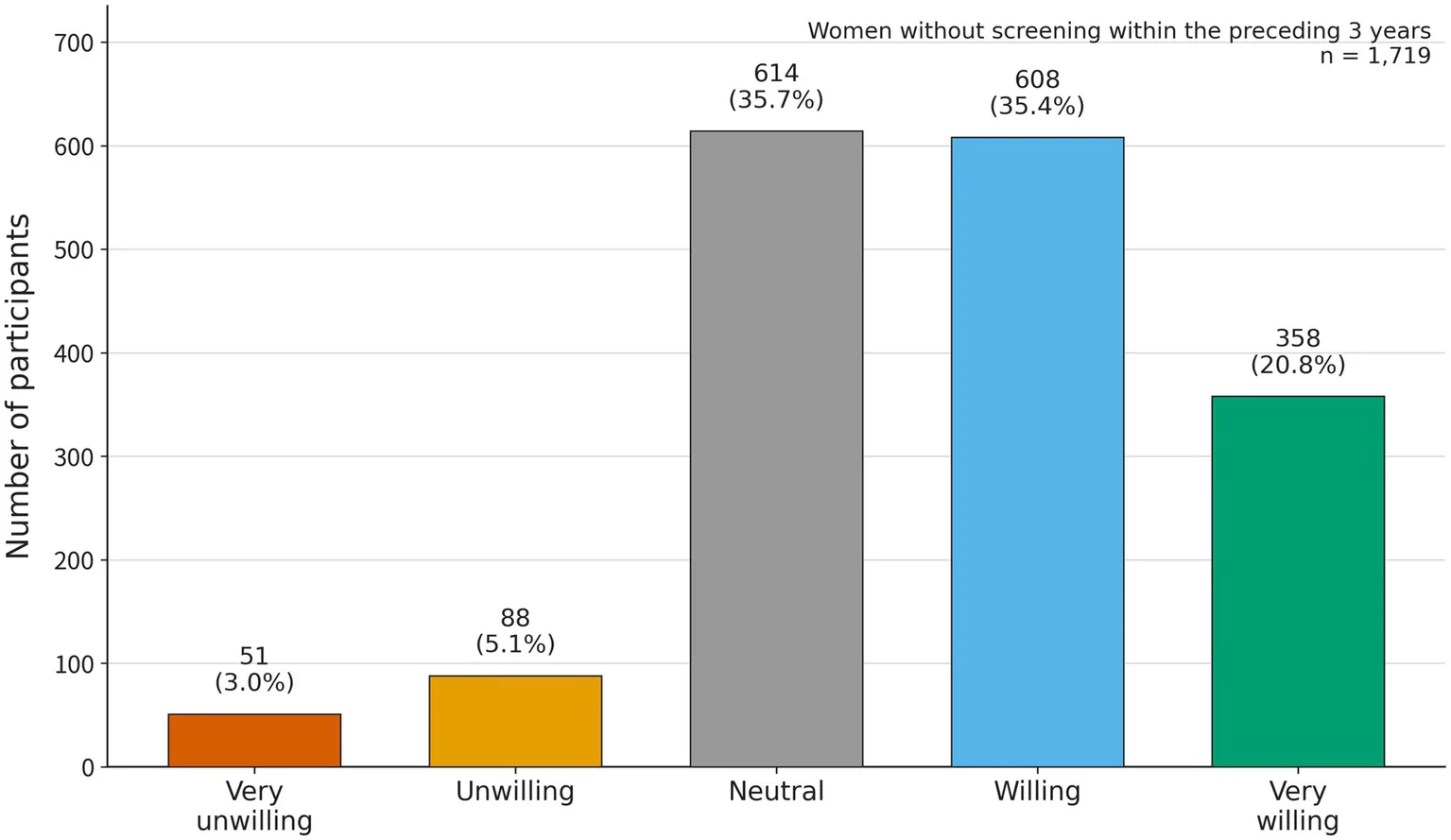
Intention to complete CCS during the current year among women not screened within the previous 3 years (*n* = 1,719). Bars show observed counts, not model predictions. Intention was assessed with the item, “I am willing to complete CCS within this year,” rated from 1 (very unwilling) to 5 (very willing). The figure is restricted to the 1,719 women without self-reported screening during the preceding 3 years. As intention and screening history were measured in the same survey, this distribution is not a prospective measure of subsequently completed screening; corresponding tabulated values are provided in Supplementary Table S3.

### 3.5 Adjusted associations with completed screening

Sequential logistic regression models were fitted to examine how the association between perceived community support and screening completion changed with progressive adjustment. In the unadjusted model, the community support score was not associated with screening completion (OR = 1.017, 95% CI 0.952–1.087; *p* = 0.607). After adjustment for sociodemographic characteristics, the estimate was essentially unchanged (aOR = 0.981, 95% CI 0.915–1.052; *p* = 0.585). Further adjustment for concurrent screening-related perceptions moved the point estimate further below the null, although the association remained statistically non-significant (aOR = 0.935, 95% CI 0.856–1.021; *p* = 0.135). The fully adjusted model is presented in Table 3, and the complete sequence of nested models is reported in Supplementary Table S4. The complete set of sociodemographic covariate estimates is reported in Supplementary Table S5.

**Table 3:** Adjusted association of overall community CC prevention support with screening completion within the previous 3 years (*n* = 3,033).

Predictor	Adjusted OR	95% CI	*p* value
Community CC prevention-support score	0.935	0.856–1.021	0.135
Knowledge of screening methods	1.184	1.102–1.271	<0.001
Belief that screening enables early detection	0.962	0.871–1.061	0.435
Screening embarrassment/discomfort	1.172	1.098–1.251	<0.001
Perceived lack of time	0.924	0.840–1.016	0.101
Screening self-efficacy	1.020	0.920–1.131	0.708

The dependent variable was self-reported screening within the preceding 3 years (1 = yes, 0 = no). The community prevention-support score is the mean of six 1–5 items, including one vaccine-service scheduling item; all displayed adjusted odds ratios represent the change in odds for a one-point increase in the relevant score. The model adjusted for age group, household registration, marital status, education, monthly income, insurance type, and parity, each entered as dummy variables. Likelihood-ratio chi-square = 123.709, df = 22, *p* < 0.001; Nagelkerke *R*-squared = 0.054. The full set of covariate estimates is reported in Supplementary Table S5. OR, odds ratio; CI, confidence interval. Concurrent assessment of screening-related measures and screening history precludes causal interpretation.

The embarrassment/discomfort item was positively associated with previous screening (aOR = 1.172, 95% CI 1.098–1.251; *p* < 0.001). This coefficient cannot be interpreted as a prospective effect of discomfort because the item and the screening history were assessed concurrently. It may reflect prior screening experience, different interpretations of the item, or reverse temporal ordering. Time barriers, belief in screening benefit, and screening self-efficacy were not independently associated with the outcome.

The item-level model produced the same overall pattern (Table 4). No individual community-support item had a positive independent association with screening completion. With Benjamini-Hochberg false discovery rate (FDR) correction for multiple testing, none of the six items remained statistically significant. Perceived availability of appointment support was inversely associated with prior screening in the mutually adjusted model (aOR = 0.881, 95% CI 0.790–0.983; *p* = 0.023), but this association did not survive FDR correction and was not observed in separate models for each item (all *p* > 0.10). The full item-level model, including screening-related covariates, is reported in Supplementary Table S6. The inverse association for expected appointment support should therefore be interpreted as a hypothesis-generating finding requiring confirmation in prospective studies.

**Table 4:** Item-level associations of perceived community support with screening completion within the previous 3 years (*n* = 3,033).

Community support item	Adjusted OR	95% CI	*p*-value
Community health promotion	0.954	0.848–1.074	0.439
Clinician recommendation	1.025	0.916–1.147	0.667
Awareness of community screening services	0.943	0.833–1.067	0.348
Trust in community health information	1.031	0.910–1.167	0.635
Expected appointment support for vaccination or screening	0.881	0.790–0.983	0.023
Vaccination-service time suitability	1.095	0.975–1.230	0.125

The dependent variable was self-reported screening within the preceding 3 years (1 = yes, 0 = no). The six community-support items were entered simultaneously; each adjusted odds ratio represents the change in odds for a one-point increase on the respective 1–5 item, conditional on the other items. The model also included the screening-related measures and sociodemographic dummy variables listed in the note to Table 3. Likelihood-ratio chi-square = 131.734, df = 27, *p* < 0.001; Nagelkerke *R*-squared = 0.057. The complete item-level model is reported in Supplementary Table S6. The inverse association for expected appointment support is exploratory and, in this cross-sectional design, cannot establish temporal direction or causation.

## 4 Discussion

This cross-sectional study examined whether perceived community CC prevention support was associated with completed CCS among women living in Shenzhen. 43.3% of women reported screening within the preceding 3 years. More than half of unscreened women expressed willingness to CCS during the current year. Perceived community prevention support showed good internal consistency but was not independently associated with completed screening after adjustment. Knowledge of CCS methods was associated with prior screening. The inverse association between expected appointment support and previous CCS was exploratory. It cannot be assigned a temporal direction. These results point to a gap between favorable perceptions, stated readiness, and completed screening.

### 4.1 Interpretation of the main findings

The null cross-sectional association does not mean community health services lack a role in CC prevention (31). The questionnaire captured perceived conditions: promotion, recommendation, awareness, trust, anticipated appointment assistance, and vaccination-service scheduling (32). It did not capture documented invitations, appointments offered, attendance, result delivery, or post-abnormal referral (33). Favorable perceptions may coexist with missed opportunities at any later step (28). Women may complete screening through hospital or employer-based routes without recalling community support (34). Thus, the result speaks to the limited ability of perceived support to differentiate prior screening in this sample. Direct comparisons with other surveys are hampered by differences in sampling, age range, and screening-interval definitions. This estimate describes the sample. It is not a benchmark.

Knowing CCS methods mattered more than a general belief in early detection (28). Believing that screening can detect disease early reflects a broad preventive attitude. Knowing the available screening methods is closer to operational knowledge: what test may be offered, where it can be obtained, and what attendance involves (14). The association with prior screening could reflect the influence of such knowledge, but it could also arise because women who have been screened learned about the methods during care. Either interpretation supports a practical distinction between generic health messaging and information that helps women navigate a specific service pathway (20).

The cross-sectional design shapes how we interpret each of these associations. Because perceived support, CCS-related beliefs, and CCS history were measured at the same time, we cannot determine whether favorable perceptions preceded screening, followed screening, or arose from other unmeasured factors. A woman who has recently completed CCS may rate community support more favorably because she remembers the process positively, or because she received help that she now recognizes as support. Conversely, a woman who has not been screened may rate support less favorably because she lacks firsthand experience, or more favorably because she hopes services will be there when she needs them (35). The same reasoning applies to the appointment-support item, where the inverse association could reflect either an unmet need among unscreened women or a post-hoc rationalization among screened women who no longer perceive a need for booking assistance (36). These alternative explanations are equally plausible given the data. The results therefore generate hypotheses about possible relationships, but they cannot confirm the direction or nature of those relationships (37).

The inverse association for appointment support is exploratory. It does not support a causal interpretation. This was an exploratory finding in a cross-sectional design, and the association did not survive correction for multiple testing. It may reflect reverse temporal ordering, unmet navigation needs, multicollinearity, suppression effects, or chance. This hypothesis-generating observation should be tested in future studies with prospective designs and documented service contacts. Women without previous screening may anticipate a greater need for booking assistance than women who have already navigated the process. The observed aOR below 1.0 is therefore compatible with reverse temporal ordering, differential interpretation of the item, or an unmet need for navigation. It does not indicate that providing appointment assistance would reduce screening (38). Future studies with prospective designs and documented service contacts should test this hypothesis (39).

The unadjusted differences by marital status, education, income, and insurance type were statistically detectable but small in magnitude. Their attenuation in the adjusted model is consistent with the limited variation explained by the model. Within this metropolitan sample, measured sociodemographic factors did not account for most differences in screening completion.

### 4.2 Practical implications for community health service delivery

The findings point to several practical directions for how community services organize and evaluate their screening programs, even though the study did not test specific interventions. In Shenzhen, where screening is often opportunistic, offered when women present for other reasons, the null association between perceived support and completed screening suggests that favorable perceptions alone may not reliably indicate whether women have completed the screening pathway. For community health managers, this implies that patient satisfaction surveys or general awareness campaigns, while valuable for other purposes, are not adequate proxies for screening uptake (40, 41). A more actionable step is to shift monitoring from perceived support to documented service contacts. Prior literature suggests that screening programs need a traceable pathway from eligibility to completed care (41). Program monitoring should distinguish the following: the identification of eligible women; the delivery of invitations; the contact method; the offer of an appointment; the confirmation of an appointment; attendance; the screening modality; the communication of results; and referral or follow-up after an abnormal result (42). These steps reflect the service continuum described in WHO guidance and permit assessment of where women disengage (43).

In Shenzhen, where electronic health records are increasingly available, building or strengthening a screening registry that records these contact points is a feasible program improvement (44). The implementation of such a system would facilitate the provision of a more useful performance measure than that afforded by perceived support alone. This system would also facilitate the identification of women who received an invitation but did not make a booking, those who made a booking but did not attend, or those who completed testing but did not receive follow-up (45). In order to ensure the protection of personal privacy, it would be necessary to implement comprehensive data governance procedures that are explicit in nature. It is also imperative to consider the necessity of coordination among a range of healthcare providers, including community centers, hospitals, laboratories, and gynecology clinics (46). Such a registry would help move from opportunistic to more systematic screening, as emphasized in WHO guidance (47).

Beyond registries, the findings point to several specific actions that community health services could take within existing resources. First, community health centers could review their current invitation practices to determine whether they systematically reach all eligible women or rely primarily on opportunistic offers during unrelated visits. Second, they could introduce a simple tracking mechanism, even a spreadsheet, to record whether women who express willingness to screen actually book and attend an appointment. This would convert the observed gap between stated readiness and completed screening from a descriptive finding into an actionable performance indicator. Third, they could test low-intensity navigation for the sizable group of willing-but-unscreened women identified in this sample. Examples include a brief follow-up phone call after a missed opportunity, a text message with a booking link, or a timed reminder before an upcoming appointment. These actions do not require new staff or complex systems (48, 49). They require a shift in how community services define their CCS role, from providing information to ensuring completion. The findings suggest that perceived support, while favorable, is not a reliable signal that this shift has occurred.

The significant proportion of unscreened women who reported a willingness or neutrality suggests a group for whom low-intensity navigation may be relevant. The service components under discussion may include, but are not limited to, a personally addressed invitation, a concise explanation of available screening tests, a direct appointment link or telephone booking option, reminder messages prior to the appointment, flexible clinic hours, and the option to reschedule after a missed visit. The provision of these components as recorded actions, as opposed to inferring them from general perceptions, would serve to enhance accountability (19, 20, 50). However, we emphasize that the specific interventions mentioned (electronic registries, active navigation, reminders, and flexible appointments) were not directly evaluated in this study. They are presented as promising avenues for future implementation research rather than as evidence-based interventions supported by the present findings (20, 37, 47). Their efficacy should be evaluated against verified attendance or completed testing, not against stated intention (19).

The descriptive pattern, with regard to education, income and insurance type, also supports the notion of proportionate service design. That is to say, it is possible to tailor the intensity of support to the level of need (51). The absolute differences across screening-status groups were minimal, and the present data do not establish that any social characteristic caused non-attendance. However, women with lower educational attainment, constrained income, limited insurance coverage, inflexible work schedules, or caregiving responsibilities may require clearer information, shorter booking procedures, and service times that reduce opportunity costs (36). Community health services have the capacity to utilize routine contact points in order to offer targeted reminders and navigation without treating these women as a homogeneous or inherently unwilling group (49).

### 4.3 Implications for research and implementation evaluation

It is recommended that future studies differentiate between perceived support, delivered support, and completed service use as separate constructs. A prospective cohort could be enrolled at the point of documented screening invitation and recorded as to whether or not navigation was received, an appointment was booked, testing was attended, results were received, and any indicated follow-up was completed. This design would provide a more comprehensive and clearer understanding of the sequence of service contact and outcome than a retrospective cross-sectional survey (52, 53).

A pragmatic trial could pose the following question: compared with standard education, does a documented invitation-and-navigation pathway increase the completion of screening? In the event of the intervention being delivered through routine facility workflows, a cluster design at the community health service center level would be deemed appropriate (27). The primary outcome should be verified screening completion rather than stated intention; secondary outcomes could include time to attendance, missed appointments, result communication, and completion of referral after an abnormal finding (54).

It is important to note that key determinants were not captured in the study, including a history of abnormal results, screening modality, test cost, clinician recommendation intensity and timing, workplace flexibility, childcare responsibilities, travel time, appointment availability, previous screening experience, and the quality of result communication. The determinants in question can be categorized into two distinct groups: individual and community- or clinic-level. Multilevel studies that integrate individual survey data with facility records and local service indicators would offer a more complete account of screening utilization.

### 4.4 Strengths and limitations

This study has several strengths. It used a large age-eligible sample, distinguished completed screening from current plans and intention, assessed internal consistency of the six-item perceived support measure, reported effect sizes alongside significance tests, and presented both composite-score and exploratory item-level models. These strengths reinforce the main findings. They do not, however, compensate for the cross-sectional design or the reliance on self-report, which are addressed in the limitations below.

The cross-sectional design warrants particular emphasis because it affects the interpretation of every association reported in this study. The data cannot support statements about what causes screening, what follows from screening, or what mediates the two. The association between CCS-method knowledge and prior screening could mean that knowledge facilitates screening, but it could also mean that women acquire knowledge through CCS, or that a third factor, such as general health engagement, produces both (55, 56). Similarly, the absence of an association between perceived community support and screening completion could indicate that perceptions do not drive screening, or it could indicate that perceptions are shaped by screening experience in ways that obscure the relationship. Without longitudinal data, we cannot distinguish these possibilities. This limitation is not unique to this study, but it is particularly consequential for interpreting null associations. A null association in cross-sectional data may reflect a true absence of relationship, a relationship that exists but is masked by concurrent measurement, or a relationship that operates in opposite directions for different subgroups (53). The present findings should therefore be used to inform the design of prospective studies, not to conclude that perceived community support is irrelevant to screening behavior.

The findings should be interpreted in light of several limitations. First, all principal variables were measured concurrently and were self-reported. Temporal ordering between perceived support, screening-related beliefs, and screening history cannot be established. Reverse causation, recall error, social-desirability bias, and post-hoc interpretation may have affected the observed associations. This limitation is especially important for expected appointment support, which should be interpreted only as an exploratory cross-sectional finding.

Second, the outcome did not specify screening modality, exact test date, clinical indication, test result, or follow-up after an abnormal result. The study did not collect records of invitation, appointment scheduling, attendance, result delivery, or referral. The exposure was therefore a perceived composite that included support for both vaccination and screening, as well as an item on vaccination-service scheduling; it was not a direct measure of delivered screening services (19).

Third, recruitment used convenience sampling through participating community health service centers and gynecology clinics. Women who could access these facilities, used mobile phones, or were already engaged with health services may be overrepresented. Importantly, this recruitment strategy may systematically exclude marginalized women, including those with limited mobility, low health literacy, language barriers, caregiving responsibilities, inflexible work schedules, or financial constraints, who may not routinely visit health facilities and who may face the greatest barriers to screening. The findings should not be generalized as population-level screening coverage for Shenzhen or to areas with different service arrangements (57, 58).

Fourth, additional limitations should be acknowledged. The non-probability convenience sample and the fact that the total number of women approached is not reported (precluding assessment of nonresponse bias) limit generalizability. Self-reported screening history may be subject to recall error and social-desirability bias; we did not verify screening through medical records or distinguish screening modality (HPV testing vs. cytology) or screening from diagnostic examinations performed for symptoms. Common-method bias may have inflated associations between concurrent measures because all data were collected through the same survey instrument. The long recruitment period (August 2023 to April 2026, approximately 33 months) and potential secular changes in screening programs, such as updated guidelines, new funding, or changes in HPV vaccination availability, may have introduced temporal heterogeneity not captured in the analysis. The three-year retrospective window varied across recruitment dates; women recruited later had a window extending further back in calendar time, which could affect recall or exposure to different policy environments.

Fifth, the questionnaire did not collect information on sexual activity status. CCS is recommended only for sexually active women, and the inclusion of women without sexual activity may have influenced the observed screening prevalence estimates. Nonetheless, all participants were recruited from clinical settings where CCS is typically offered to sexually active women, which partially mitigates this concern.

Sixth, screening in this setting was largely opportunistic, offered to women already presenting for other reasons. This may have enriched the sample with women already engaged with health services and limited the ability to assess barriers faced by women who do not routinely seek care.

Finally, the model explained only a limited proportion of variation in screening completion, indicating that relevant determinants were unmeasured.

## 5 Conclusion

In this Shenzhen sample, perceived community CC prevention support was favorable but was not independently associated with self-reported screening completed within the preceding 3 years. Knowledge of screening methods was associated with prior screening, while the appointment-support result remained exploratory. The public health implication is not that community services are unimportant. The findings suggest that programs should shift their focus from measuring perceptions to tracking documented service contacts: invitations, bookings, attendance, result communication, and follow-ups. Whether active navigation pathways increase verified screening completion beyond standard health education is a question for prospective studies that can establish temporal order and distinguish perceived support from delivered support.

## Data Availability

The raw data supporting the conclusions of this article will be made available by the authors, without undue reservation.

## Footnotes

1
www.wjx.cn
