# Supplementary material for: Perceived community cervical cancer prevention support and cervical cancer screening utilization among women in Shenzhen, China: a cross-sectional study
Source: Front Public Health. 2026 Sep 23;14:1964812. doi: 10.3389/fpubh.2026.1964812 (PMC13642793; doi:10.3389/fpubh.2026.1964812)
Supplement: Supplementary file 1 [file Supplementary_file_1.docx]

Supplementary Material S1. Bilingual community survey questionnaire

# Translation note

This document presents the Chinese questionnaire and an English translation prepared for scientific review. The Chinese version is the source instrument. “Household registration” refers to the Chinese *hukou* administrative registration system. “Employee medical insurance” and “resident medical insurance” refer to the corresponding Chinese social health-insurance schemes.

# Participant information / 受访者说明

**Chinese:**  尊敬的女士：您好！我们正在进行一项关于 HPV 疫苗犹豫与宫颈癌早筛防治的社区多层级干预体系研究。问卷采用匿名方式，约需 15 分钟完成。所有信息仅用于学术研究并严格保密。您的参与完全自愿。

**English:**  Dear participant: We are conducting a study of HPV vaccine hesitancy and community-based multilevel interventions for early cervical cancer prevention and control. The anonymous questionnaire takes approximately 15 minutes. Information will be used only for academic research and will be kept confidential. Participation is voluntary.

# Section 1. Sociodemographic characteristics / 第一部分：基本信息

| **No.** | **Chineseitem** | **Englishtranslation** | **Responseoptions/coding** |
| --- | --- | --- | --- |
| 1 | 年龄 | Age | <18; 18-26; 27-37; 38-48; 49-65; >65 years |
| 2 | 户籍 | Household registration (*hukou*) | Shenzhen household registration; non-Shenzhen household registration |
| 3 | 婚姻状况 | Marital status | Never married; married; divorced/widowed |
| 4 | 教育程度 | Educational attainment | Junior high school or below; high school/technical secondary school; college diploma; bachelor's degree or above |
| 5 | 职业 | Occupation | Student; employee in an enterprise; government/public institution employee; self-employed; homemaker; other |
| 6 | 个人月收入 | Personal monthly income | <5,000; 5,000-9,999; 10,000-19,999; >=20,000 yuan |
| 7 | 医保类型 | Health insurance type | No insurance; resident medical insurance; employee medical insurance; commercial insurance |
| 8 | 生育情况 | Childbearing status | No childbirth; childbirth history (number of children reported) |

# Section 2. Basic knowledge / 第二部分：基础认知测量

**Chinese instruction:**  请根据了解程度选择：1=完全不了解，5=非常了解。

**English instruction:** Please rate your level of knowledge: 1=not at all familiar; 5=very familiar.

| **No.** | **Chineseitem** | **Englishtranslation** |
| --- | --- | --- |
| K1 | 您对 HPV 病毒（人乳头瘤病毒）的了解程度 | How familiar are you with human papillomavirus (HPV)? |
| K2 | 您对 HPV 疫苗（宫颈癌疫苗）的了解程度 | How familiar are you with HPV vaccines (cervical cancer vaccines)? |
| K3 | 您对宫颈癌筛查方法（如 TCT、HPV 检测）的了解程度 | How familiar are you with cervical cancer screening methods, such as cytology/TCT and HPV testing? |
| K4 | 您知道 HPV 疫苗可以预防宫颈癌吗？ | Do you know that HPV vaccination can prevent cervical cancer? |
| K5 | 您知道宫颈癌筛查可以早期发现病变吗？ | Do you know that cervical cancer screening can detect abnormalities early? |
| K6 | 您知道深圳市有哪些 HPV 疫苗接种点？ | Do you know where HPV vaccination services are available in Shenzhen? |
| K7 | 您知道深圳市宫颈癌筛查的惠民政策吗？ | Do you know about Shenzhen policies that support access to cervical cancer screening? |
| K8 | 您知道 HPV 疫苗的最佳接种年龄吗？ | Do you know the recommended age for HPV vaccination? |

*Note: All Section 2 items use the 1-5 knowledge scale specified above.*

# Section 3. Health belief measures / 第三部分：健康信念模型测量

**Chinese instruction:** 请根据同意程度选择：1=非常不同意，5=非常同意。

**English instruction:** Please indicate your agreement: 1=strongly disagree; 5=strongly agree.

| **Code** | **Chineseitem** | **Englishtranslation** |
| --- | --- | --- |
| H1 | 我认为自己有可能感染 HPV 病毒 | I believe that I may become infected with HPV. |
| H2 | 我认识的女性中有人感染过 HPV 或患宫颈癌 | Among women I know, someone has had HPV infection or cervical cancer. |
| H3 | 像我这个年龄的女性，感染 HPV 的风险不低 | Women of my age have a meaningful risk of HPV infection. |
| H4 | 我认为宫颈癌是一种严重的疾病 | I consider cervical cancer a serious disease. |
| H5 | 如果患上宫颈癌，会对我的生活造成严重影响 | Cervical cancer would have a serious impact on my life. |
| H6 | 宫颈癌的治疗过程会很痛苦且花费巨大 | Treatment for cervical cancer would be painful and expensive. |
| H7 | 接种 HPV 疫苗能有效降低宫颈癌风险 | HPV vaccination can effectively reduce the risk of cervical cancer. |
| H8 | 定期筛查能早期发现宫颈问题 | Regular screening can detect cervical problems early. |
| H9 | 早期发现的宫颈病变治愈率很高 | Cervical lesions detected early have a high cure rate. |
| H10 | 接种疫苗的费用对我来说有负担 | The cost of vaccination is a burden to me. |
| H11 | 做筛查让我感到尴尬或不适 | Cervical cancer screening makes me feel embarrassed or uncomfortable. |
| H12 | 我没有时间去接种疫苗或做筛查 | I do not have time to receive vaccination or undergo screening. |
| H13 | 我能安排好时间完成疫苗接种全程 | I can arrange my time to complete the full vaccination schedule. |
| H14 | 我有信心能克服困难去做宫颈癌筛查 | I am confident that I can overcome difficulties to undergo cervical cancer screening. |
| H15 | 我知道如何获取准确的 HPV 疫苗信息 | I know how to obtain accurate information about HPV vaccination. |

*Note: All Section 3 items use the 1-5 agreement scale specified above.*

# Section 4. Vaccine hesitancy measures / 第四部分：基于 3C 模型的疫苗犹豫测量

**Chinese instruction:** 请根据同意程度选择：1=非常不同意，5=非常同意。

**English instruction:** Please indicate your agreement: 1=strongly disagree; 5=strongly agree.

| **Code** | **Chineseitem** | **Englishtranslation** |
| --- | --- | --- |
| C1 | 我觉得自己很健康，不太可能得宫颈癌 | I consider myself healthy and unlikely to develop cervical cancer. |
| C2 | 宫颈癌离我很遥远，不需要特别关注 | Cervical cancer feels remote from my life and does not require special attention. |
| C3 | 我的生活方式健康，不需要额外预防 | My lifestyle is healthy, so I do not need additional prevention. |
| C4 | 我相信 HPV 疫苗是安全有效的 | I believe that HPV vaccines are safe and effective. |
| C5 | 我担心疫苗有严重的副作用 | I am concerned that vaccines may have serious side effects. |
| C6 | 我认为疫苗预防宫颈癌的效果是确切的 | I believe that vaccines have a definite effect in preventing cervical cancer. |
| C7 | 我信任医生关于接种疫苗的建议 | I trust physicians' recommendations about vaccination. |
| C8 | 我信任国内疫苗生产企业的产品质量 | I trust the quality of vaccines produced by domestic manufacturers. |
| C9 | 我信任政府部门的疫苗监管体系 | I trust the government system for vaccine regulation. |
| C10 | 疫苗接种点离我家/单位很方便 | Vaccination sites are conveniently located near my home or workplace. |
| C11 | 预约接种或筛查的程序很简单 | The process for booking vaccination or screening is simple. |
| C12 | 我能方便地获取疫苗/筛查的准确信息 | I can easily obtain accurate information about vaccination and screening. |

*Note: All Section 4 items use the 1-5 agreement scale specified above.*

**Section 5. Social cognition and community support / 第五部分：社会认知与社区支持测量**

**Chinese instruction:**  1-5 表示同意程度：1=非常不同意，5=非常同意。

**English instruction:**  Please indicate your agreement: 1=strongly disagree; 5=strongly agree.

| **Code** | **Chineseitem** | **Englishtranslation** |
| --- | --- | --- |
| S1 | 我的家人支持我接种 HPV 疫苗 | My family supports my receiving HPV vaccination. |
| S2 | 我的朋友/同事中有不少人接种了 HPV 疫苗 | Many of my friends or colleagues have received HPV vaccination. |
| S3 | 我所在的社区经常宣传宫颈癌防治知识 | My community frequently promotes knowledge about cervical cancer prevention and control. |
| S4 | 社区医生曾向我推荐过 HPV 疫苗或筛查 | A community physician has recommended HPV vaccination or screening to me. |
| S5 | 我知道社区有宫颈癌筛查服务 | I know that cervical cancer screening services are available in my community. |
| S6 | 社区提供的健康信息是可信的 | Health information provided by the community is trustworthy. |
| S7 | 如果需要，社区工作人员会帮助我预约接种/筛查 | If needed, community staff would help me book vaccination or screening. |
| S8 | 社区的疫苗接种服务时间安排很合理 | The scheduling of community vaccination services is reasonable. |

*Note: All Section 5 items use the 1-5 agreement scale specified above. In the present screening-service analysis, S3-S8 were used to form the community cervical cancer prevention-support score. This score includes one item on vaccination-service scheduling and should not be interpreted as a direct measure of delivered screening services.*

# Section 6. Preventive behavior and intention / 第六部分：行为现状与意向

| **No.** | **Chineseitem** | **Englishtranslation** | **Responseoptions** |
| --- | --- | --- | --- |
| B1 | 您是否接种过 HPV 疫苗？ | Have you received HPV vaccination? | Completed full vaccination; vaccinated but not completed; not vaccinated but plans vaccination within 6 months; not vaccinated and no plan |
| B2 | 您过去 3 年内是否做过宫颈癌筛查？ | Have you undergone cervical cancer screening within the past 3 years? | Yes; no, but plan to do so within this year; no, and no plan |
| B3 | 如果您未接种/未筛查，最主要的原因是？（单选） | If you have not been vaccinated or screened, what is the main reason? (select one) | Cost is too high; concerned about safety/side effects; no time; unnecessary; do not know where to obtain vaccination/screening; other |
| I1 | 我愿意在未来 6 个月内接种 HPV 疫苗 | I am willing to receive HPV vaccination within the next 6 months. | 1=very unwilling to 5=very willing |
| I2 | 我愿意在今年内完成宫颈癌筛查 | I am willing to complete cervical cancer screening within this year. | 1=very unwilling to 5=very willing |
| I3 | 我愿意参加社区组织的宫颈癌防治讲座 | I am willing to attend a community-organized cervical cancer prevention lecture. | 1=very unwilling to 5=very willing |
| I4 | 我愿意推荐亲友接种 HPV 疫苗 | I am willing to recommend HPV vaccination to relatives and friends. | 1=very unwilling to 5=very willing |
| B4 | 信息获取偏好（多选） | Preferred channels for obtaining vaccination/screening information (multiple response) | Community notice board; WeChat official account; face-to-face physician consultation; family/friends; short-video platform; community health lecture; other |

# Section 7. Open-ended questions / 第七部分：开放性问题

| **No.** | **Chineseitem** | **Englishtranslation** |
| --- | --- | --- |
| O1 | 您认为社区在推广 HPV 疫苗接种和宫颈癌筛查方面，最需要改进的是什么？ | What most needs to be improved in your community's promotion of HPV vaccination and cervical cancer screening? |
| O2 | 您对 HPV 疫苗或宫颈癌筛查还有什么疑问或担忧？ | What questions or concerns do you still have about HPV vaccination or cervical cancer screening? |

*Note: Open-ended responses were optional. This bilingual version is intended as supplementary material for international readers and should be checked against the final ethics-approved source questionnaire before submission.*
