# Supplementary material for: Perceived community cervical cancer prevention support and cervical cancer screening utilization among women in Shenzhen, China: a cross-sectional study
Source: Front Public Health. 2026 Sep 23;14:1964812. doi: 10.3389/fpubh.2026.1964812 (PMC13642793; doi:10.3389/fpubh.2026.1964812)
Supplement: Supplementary file 2 [file Table_1.docx]

Supplementary materials

# Supplementary Table S1. Internal consistency of support measures

| **Scale** | **Items** | **Cronbach's alpha** | **Interpretation** |
| --- | --- | --- | --- |
| Interpersonal social support | 2 | 0.764 | Acceptable |
| Community cervical cancer prevention support | 6 | 0.918 | Good |
| Eight-item social and community support scale | 8 | 0.934 | Excellent |

*Note: This table is cited in Results section 3.3. Interpersonal social support comprised family support for HPV vaccination and peer vaccination norms. Community cervical cancer prevention support comprised community promotion, clinician recommendation, awareness of screening services, trust in community information, expected appointment support for vaccination or screening, and vaccination-service time suitability. The eight-item scale combined the two interpersonal and six community items. Alpha values should be interpreted alongside the number and conceptual breadth of items.*

# Supplementary Table S2. Individual community cervical cancer prevention-support items by screening status (n=3,033)

| **Community prevention-support component** | **No screening/no plan, median (P25, P75)** | **Not screened/plans this year, median (P25, P75)** | **Screened within 3 years, median (P25, P75)** | **H** | ***p* value** | **Eta-squared** |
| --- | --- | --- | --- | --- | --- | --- |
| Community health promotion | 4.00 (3.00, 5.00) | 4.00 (3.00, 5.00) | 4.00 (3.00, 5.00) | 4.946 | 0.084 | 0.002 |
| Clinician recommendation | 4.00 (3.00, 4.00) | 4.00 (3.00, 5.00) | 4.00 (3.00, 5.00) | 0.712 | 0.701 | <0.001 |
| Awareness of community screening services | 4.00 (3.00, 5.00) | 4.00 (4.00, 5.00) | 4.00 (3.00, 5.00) | 4.720 | 0.094 | 0.002 |
| Trust in community health information | 4.00 (3.00, 4.00) | 4.00 (3.00, 4.00) | 4.00 (3.00, 4.00) | 0.635 | 0.728 | <0.001 |
| Expected appointment support for vaccination or screening | 4.00 (3.00, 4.00) | 4.00 (3.00, 4.00) | 4.00 (3.00, 4.00) | 13.022 | 0.001 | 0.004 |
| Vaccination-service time suitability | 4.00 (3.00, 4.00) | 4.00 (3.00, 4.00) | 4.00 (3.00, 4.00) | 0.610 | 0.737 | <0.001 |

*Note: This table is cited in Results section 3.3 through the note to Table 2. All items were rated from 1 (strongly disagree) to 5 (strongly agree), with higher scores indicating greater perceived support. Values are median (P25, P75). Kruskal-Wallis tests compared the three independent screening-status groups. Eta-squared was calculated as H/(N-1). The appointment-support item asks about assistance with booking vaccination or screening, and the service-time item asks about vaccination-service scheduling. These measures were assessed concurrently with screening history and do not establish temporal ordering or causation.*

# Supplementary Table S3. Screening intention among women not screened during the previous 3 years (n=1,719)

| **Intention score for screening within the current year** | **n** | **%** |
| --- | --- | --- |
| 1, very unwilling | 51 | 3.0 |
| 2, unwilling | 88 | 5.1 |
| 3, neutral | 614 | 35.7 |
| 4, willing | 608 | 35.4 |
| 5, very willing | 358 | 20.8 |

*Note: This table is cited in Results section 3.4. The item asked, “I am willing to complete cervical cancer screening within this year.” Percentages use the 1,719 age-eligible women who had not completed screening during the preceding 3 years as the denominator. A planned screening response in the screening-status item and a Likert intention-score measure were related but non-identical constructs. As intention was measured at the same survey time point, these values do not represent subsequently completed screening.*

# Supplementary Table S4. Complete adjusted logistic regression model for screening completion (n=3,033)

| **Predictor** | **Adjusted OR** | **95% CI** | ***p* value** |
| --- | --- | --- | --- |
| Community cervical cancer prevention-support score | 0.935 | 0.856-1.021 | 0.135 |
| Knowledge of screening methods | 1.184 | 1.102-1.271 | <0.001 |
| Belief that screening enables early detection | 0.962 | 0.871-1.061 | 0.435 |
| Screening embarrassment/discomfort | 1.172 | 1.098-1.251 | <0.001 |
| Perceived lack of time | 0.924 | 0.840-1.016 | 0.101 |
| Screening self-efficacy | 1.020 | 0.920-1.131 | 0.708 |
| Age 27-37 vs 18-26 years | 0.904 | 0.729-1.122 | 0.361 |
| Age 38-48 vs 18-26 years | 0.830 | 0.617-1.116 | 0.217 |
| Age 49-65 vs 18-26 years | 0.893 | 0.589-1.355 | 0.595 |
| Non-Shenzhen vs Shenzhen household registration | 1.072 | 0.925-1.242 | 0.357 |
| Never married vs married | 1.180 | 0.994-1.401 | 0.059 |
| Divorced/widowed vs married | 1.306 | 0.952-1.793 | 0.098 |
| College diploma vs junior high school or below | 0.651 | 0.437-0.972 | 0.036 |
| Bachelor's degree or above vs junior high school or below | 0.636 | 0.425-0.953 | 0.028 |
| High school/technical secondary vs junior high school or below | 0.718 | 0.477-1.081 | 0.112 |
| Income 10,000-19,999 vs <5,000 yuan | 0.806 | 0.649-1.002 | 0.052 |
| Income 5,000-9,999 vs <5,000 yuan | 0.947 | 0.782-1.147 | 0.579 |
| Income >=20,000 vs <5,000 yuan | 0.813 | 0.609-1.085 | 0.159 |
| Resident insurance vs commercial insurance | 1.055 | 0.761-1.462 | 0.747 |
| No insurance vs commercial insurance | 0.965 | 0.642-1.451 | 0.864 |
| Employee insurance vs commercial insurance | 1.344 | 0.981-1.840 | 0.065 |
| Parous vs nulliparous | 0.997 | 0.813-1.222 | 0.974 |

*Note: This table is cited in Results section 3.5. The outcome was self-reported cervical cancer screening within the preceding 3 years (1=yes, 0=no). Odds ratios are mutually adjusted for all predictors in the table. For 1-5 screening-related measures, estimates represent the change in odds for a one-point increase; reference categories for categorical covariates are shown after “vs”. The model used the complete age-eligible analytic sample (n=3,033). Likelihood-ratio chi-square=123.709, df=22, p<0.001; Nagelkerke R-squared=0.054. The cross-sectional design does not establish temporal order or causation. OR, odds ratio; CI, confidence interval.*

**Supplementary Table S5. Complete item-level logistic regression model for screening completion (n=3,033)**

| **Predictor** | **Adjusted OR** | **95% CI** | ***p* value** |
| --- | --- | --- | --- |
| Community health promotion | 0.954 | 0.848-1.074 | 0.439 |
| Clinician recommendation | 1.025 | 0.916-1.147 | 0.667 |
| Awareness of community screening services | 0.943 | 0.833-1.067 | 0.348 |
| Trust in community health information | 1.031 | 0.910-1.167 | 0.635 |
| Expected appointment support for vaccination or screening | 0.881 | 0.790-0.983 | 0.023 |
| Vaccination-service time suitability | 1.095 | 0.975-1.230 | 0.125 |
| Knowledge of screening methods | 1.175 | 1.094-1.262 | <0.001 |
| Belief that screening enables early detection | 0.965 | 0.874-1.065 | 0.476 |
| Screening embarrassment/discomfort | 1.162 | 1.087-1.241 | <0.001 |
| Perceived lack of time | 0.923 | 0.840-1.015 | 0.100 |
| Screening self-efficacy | 1.022 | 0.922-1.134 | 0.676 |

*Note: This table is cited in Results section 3.5. The outcome was self-reported cervical cancer screening within the preceding 3 years (1=yes, 0=no). All six community-support items were entered simultaneously; their odds ratios represent the change in odds for a one-point increase on the respective 1-5 item. The model additionally adjusted for the screening-related measures, age group, household registration, marital status, education, monthly income, insurance type, and parity. Sociodemographic covariate coefficients are not repeated because they are presented in Supplementary Table S2 and were not the focus of this exploratory item-level analysis. The complete age-eligible analytic sample (n=3,033) was used. Likelihood-ratio chi-square=131.734, df=27, p<0.001; Nagelkerke R-squared=0.057. Cross-sectional associations do not establish temporal order or causation. OR, odds ratio; CI, confidence interval.*
